# ASPHER Statement: A New Public Health Curriculum for a “New Normal”

**DOI:** 10.3389/phrs.2023.1606539

**Published:** 2023-11-15

**Authors:** Mary Codd, Henrique Barros, Nadav Davidovitch, Oliver Razum, Mzwandile Mabhala, Polychronis Kostoulas, Mirjana Kujundžic Tiljak, Karolina Lyubomirova, Karl F. Conyard, Olalekan Popoola, Maly Morshad Ahmad, Lore Leighton, Robert Otok, Carlo Signorelli

**Affiliations:** ^1^ Association of Schools of Public Health in the European Region (ASPHER), Brussels, Belgium; ^2^ School of Public Health, Physiotherapy and Sports Science, University College Dublin, Dublin, Ireland; ^3^ Institute of Public Health (ISPUP), University of Porto, Porto, Portugal; ^4^ School of Public Health, Ben Gurion University of the Negev, Beer-Sheva, Israel; ^5^ School of Public Health, Bielefeld University, Bielefeld, Germany; ^6^ Faculty of Health and Social Care, University of Chester, Chester, United Kingdom; ^7^ Department of Public and One Health, School of Health Sciences, University of Thessaly, Karditsa, Greece; ^8^ Andrija Štampar School of Public Health, School of Medicine, University of Zagreb, Zagreb, Croatia; ^9^ Faculty of Public Health, Medical University of Sofia, Sofia, Bulgaria; ^10^ School of Hygiene and Preventive Medicine, University Vita-Salute San Raffaele, Milano, Italy

**Keywords:** new normal, public health, core curriculum, public health workforce, ASPHER

**Statement of the Association of Schools of Public Health in the European Region (ASPHER)**

## What Is the “New Normal”?

The constant state of change that epitomizes our existence inevitably results in new phenomena that impact individuals, professions, and society. In the context of public health (PH), the “new normal” of our time has been and is being shaped by demographic and socioeconomic shifts, scientific and technological developments, political strife, upheaval and migration, climate change and environmental degradation, food and water insecurity, and the COVID-19 pandemic. Thus, the “new normal” refers to a constant state of risk necessitating a state of “preparedness” for change which cannot be fully contained or overcome but which calls for recognition and regulation [1]. The Association of Schools of Public Health in the European Region (ASPHER) recognizes this “new normal” for PH and the challenges it poses for PH education, training, and practice.

The COVID-19 pandemic exacerbated an evolving series of health crises in an increasingly unstable world [2]. It highlighted issues of trust in science and expertise among the public in general, governments, politicians, and healthcare workers. It capitalized on rapid developments and dissemination of information and exposed the potential of mis and disinformation. It emphasized the need for competent leadership and informed decision-making, and it highlighted the growing inequities of our time. Any acceptance of this “new normal” as being inevitable or hopeless must be addressed, and its potential for positive change must be embraced [3].

In many respects, addressing the pandemic was basic PH, comprising epidemiology, surveillance, contact tracing, implementing interventions of isolation, and vaccination. These must be maintained and strengthened as essential PH services [2]. However, expertise and skills above what have been traditional tenets of PH are also required [4] affirming ASPHER’s endorsement of a multi-disciplinary approach, particularly for preparedness [5]. While current PH education may be constrained by conventional instructional approaches and challenges in the integration of theoretical knowledge with practical applications, essential competencies for an informed PH workforce are evolving [6].

For these reasons, ASPHER, in collaboration with the World Health Organization (WHO) Regional Office for Europe, has undertaken to review a core competencies for PH to address the “new normal” and to underpin the attainment of necessary competencies by PH graduates. This is the theme of this year’s ASPHER-member conference, the 2023 ASPHER Deans’ and Directors’ Retreat.

## What Is Needed to Prepare PH for the “New Normal”?

ASPHER, adhering to its mission to improve and protect the public by strengthening the education and training of PH professionals for practice and research, must address the competencies required of the PH workforce for the “new normal.” Previous frameworks and roadmaps [7–9] act as guides; however, we continuously seek to identify and fill gaps in skills and knowledge.

### The PH Workforce: Multidisciplinary, Interprofessional, and Cross-Sectoral

A multidisciplinary PH workforce must work in interprofessional and cross-sectoral contexts recognizing the skills, values, languages, and methodologies of other disciplines. Interventions critical to PH, such as vaccination or food regulation, rely on collaboration across specialisms, an understanding of political, societal, and ethical contexts, and the leadership to engage stakeholders, create consensuses, and make tough decisions. The pandemic disrupted every facet of existence; exposed underlying social and structural factors that influenced exposure, testing, access to healthcare, vaccination, and survival; and identified political, cultural, religious, and philosophical beliefs that impacted interventions [10].

Politics, and how it influences inequalities, is fundamental to PH. Political decisions determine funding for national health systems and health agencies, how guidelines and regulations are enforced, how vaccine and health workers are apportioned, and ultimately how morbidity and mortality vary by social status, race, gender, and ethnicity [10]. Political strife due to increasing populism, conflict and war, and distrust in science serve to amplify the need for PH professionals to engage with politics and policymakers in the interest of the health of the public [11].

### The PH Workforce: The Sustainability Agenda and Digital Advancements

Climate change affects ecosystems which impact health via altered disease patterns, food security, and natural disasters. PH professionals must be prepared for environmental threats, honing green skills, promoting sustainability, and engaging One Health strategies. PH professionals must devise holistic strategies to promote and preserve health, prevent risk, and design solutions for human, animal, and environmental wellbeing coupled with social contexts. One Health informs traditional PH disciplines with advancements in the identification and surveillance of zoonotic agents, genomics, and development of vaccines and therapeutics [12].

Developments in digital technologies offer a wealth of resources to PH for data management and research. PH professionals must be equipped with the knowledge and skills required to respond to, and use, big data and artificial intelligence [13].

### The PH Workforce Must Act Ethically

PH ethics is specific to the field, differs from bioethics approaches, and is essential to guide PH decision-making and action. The principles of PH ethics, often circumvented in emergencies as evident during the pandemic, require PH professionals to address the fundamental causes of disease justly and fairly. It ensures that interventions, policies, and programmes prioritize the wellbeing of populations while respecting individual rights and values. It addresses complex moral dilemmas and promotes fairness, transparency, and community engagement, helping prevent potential harms, inequities, and unethical practices.

In the “new normal,” PH ethics is needed more than ever to introduce solidarity for local and global questions, and to know how to practice PH, understanding the role of diversity, social justice, and communities. PH professionals must be ready to balance these tensions, grasping the dynamics of challenges and the constant need for reflection [1].

## The ASPHER Core Curriculum Supporting PH Preparedness for the “New Normal”

COVID-19 and other crises have ushered in a “new normal” as it relates to PH practice. It is incumbent upon us to evolve our teaching accordingly. Integrating the concept of the “new normal” into PH curricula is essential to equip PH graduates to effectively navigate strategies and interventions to respond to health crises in an ever-changing world, thereby protecting and promoting the health and welfare of the global population and planet. As a necessary step to a prepared PH workforce, and to ensure that ASPHER-member PH programmes are fit for purpose, the ASPHER Executive Board in collaboration with WHO has undertaken a wide-ranging review of PH curricula across 150 member schools and beyond. From these consultations, we will profile a core competencies for the “new normal” PH to be introduced at the 2023 ASPHER-member conference.

## Editorial Note

The authors are responsible and liable for the contents of this Society Statement. Society Statements in PHR are checked for clarity and approved by an Editor-in-Chief. Society Statements are not externally peer reviewed.
